# Effect of lactoferrin on enamel characteristics of primary and permanent teeth: an in-vitro study

**DOI:** 10.1186/s12903-023-03709-1

**Published:** 2023-12-11

**Authors:** Nouran Samir Mohamed Atia, Reham Ahmed El-nemr, Asmaa Ali Emam Abo-Elsoud

**Affiliations:** Department of Pediatric Dentistry, Preventive Dentistry and Dental Public Health, Faculty of Dentistry, Suez Canal University, Ismaillia, Egypt

**Keywords:** Lactoferrin, Hardness, Enamel

## Abstract

**Background:**

Lactoferrin, a glycoprotein naturally found in breast milk, is known for its bactericidal and antiviral properties, as well as its capacity to modulate the immune system; therefore, pediatricians routinely recommend it as dietary support. The objective of this study was to determine how lactoferrin oral suspension could affect the enamel surface characteristics of primary and permanent teeth.

**Methods:**

This research was conducted on 40 unidentified extracted teeth, including primary and permanent teeth. Experimental teeth were free of cracks or enamel defects, as confirmed by careful examination using a dental operating microscope. The crowns were bisected into 80 specimens and assorted into two groups based on the type of dentition. Group DM included 40 specimens of second deciduous molars, while Group PM contained 40 samples of first premolars. Each of the DM and PM specimens was subsequently split based on the type of dispersion medium into two subgroups: a control subgroup (artificial saliva) and a test subgroup (lactoferrin suspension). The specimens were immersed in lactoferrin suspension for two minutes, then kept in artificial saliva for the rest of the 24 h for 30 successive days. This is a pioneering study about the effect of orally supplemented lactoferrin on teeth; therefore, we examined enamel hardness, ultra-morphology, and mineral contents.

**Results:**

Our findings indicated a highly significant decrease (*p* < 0.01) in the microhardness of the lactoferrin subgroup in Group DM (second deciduous molars) and a significant reduction (*p* < 0.05) in the microhardness of the lactoferrin subgroup in Group PM (premolars). Calcium weight% was not statistically different (*p* > 0.05) compared with a significant decline (*p* < 0.05) in phosphorus weight% in lactoferrin subgroups in both DM and PM groups. The enamel surface of lactoferrin subgroups in both DM and PM groups was demineralized and porous, with the enamel of deciduous teeth being more affected by lactoferrin than permanent teeth.

**Conclusion:**

Lactoferrin suspension decreased the microhardness of enamel and both calcium and phosphorus weight percentages. Both dentitions exhibited erosions in the enamel surface, with primary teeth being more affected than the permanent teeth.

## Background and introduction

Lactoferrin is an iron-binding protein abundant in polymorphonuclear leukocytes, external bodily fluids, and breast milk, serving many biological purposes. Orally administered lactoferrin mediates several protective actions, including immunomodulation and anti-bacterial activity, along with specific anti-inflammatory and anticancer properties [1].

The synthesis of new bioactive compounds derived from milk proteins is a prominent component of “functional food” production. The substances obtained from milk are safe and beneficial for health. Milk proteins, particularly lactoferrin, show the broadest range of biological activity in living organisms [2–4].

Bovine lactoferrin is also a safer and more effective source of elemental iron to treat iron depletion and anemia due to iron insufficiency. Taking bovine lactoferrin orally for 30 days increased the red blood cell count, hemoglobin, serum ferritin, and total iron [5]. The potential benefits of lactoferrin opened avenues to add this nutraceutical protein to several nutritional and pharmaceutical products.

Dental erosion is an irreversible destruction of tooth structure caused by chemical acid dissolution irrelevant to plaque bacteria. Intrinsic factors, including vomiting and acid reflux, may contribute to dental erosion, similar to extrinsic variables, such as consuming food, drink, or medication [6]. Considering the difficulty of swallowing tablets, liquid oral medication is the most widely prescribed form of medication for juveniles. However, oral medications in the form of suspensions influence the hardness of enamel and change its morphological pattern [7]. Acidic preparations are required to preserve chemical stability, facilitate medicine dispersion, assure physiological compatibility, and enhance flavor. Aside from the acidic ingredients, other causes, including frequent and extended intake, consumption at sleep time or between repasts, high viscosity, and decreased salivary flow, may also raise the hazard of medication-induced dental erosion [8].

Numerous studies have been conducted on the clinical importance of enamel demineralization, focusing on how primary and permanent teeth differ in their vulnerability to enamel degradation. Primary teeth have a 50% higher mineral loss and a 30% deeper lesion, making them more vulnerable to enamel erosion [9]. Primary tooth enamel has a lower outer layer density and is less mineralized than permanent tooth enamel [10].

Previously conducted investigations have examined the impact of several popular pediatric syrups on the properties of the enamel surface. Regretfully, no studies assessed the influence of lactoferrin suspension on the properties of permanent or primary tooth enamel. Lactoferrin is accessible in tablet or sachet form [11]; hence, this pioneering research investigated the effect of orally administered lactoferrin (oral suspension form) on the enamel of primary and permanent teeth using the surface microhardness test (Vickers test) and energy dispersive X-ray spectroscopy (EDX) to measure mineral content, and an environmental scanning electron microscope (ESEM) to monitor enamel ultra-morphology. The null hypothesis was that lactoferrin suspension would not affect the enamel surface of primary or permanent teeth.

## Methods

### Specimen preparations

All the patients under 16 years old who presented for extraction at the Department of Pediatric Dentistry and the Department of Oral and Maxillofacial Surgery, Suez Canal University, had their parents or legal guardians sign the informed consent forms to employ their removed teeth for research purposes. All of the performed procedures of the present study were authorized by the Research Ethics Committee (REC) of the Faculty of Dentistry at Suez Canal University with permission number 410/2021, conforming to the World Medical Association’s Helsinki Declaration (Version 2008). Forty freshly extracted anonymous teeth were carefully explored using a dental operating microscope to confirm they were free of cracks, cavities, or restorations. Twenty teeth were sound second deciduous molars extracted due to shedding and obtained from the Department of Pediatric Dentistry at Suez Canal University. The other twenty were sound premolars removed due to orthodontic treatment and obtained from the Department of Oral and Maxillofacial Surgery at Suez Canal University. The extracted teeth were meticulously cleaned and autoclaved at 121 °C for 20 min, then stored in normal saline until the first day of the experiment.

The sample size was estimated using the independent samples T-test with a power of 95% and an effect size of 0.75, utilizing alpha (α) level of 0.05 and beta (β) level of 0.05. The estimated sample size (n) was 80, as calculated utilizing G*Power version 3.1.9.2 [12].

All crowns of the second deciduous molars and the premolars were separated from roots at the cementoenamel junction by a sharp disc. Then, the crowns were bisected mesio-distally using the same tool to obtain buccal and lingual halves, resulting in 80 teeth specimens. The specimens were inserted horizontally in the acrylic mold (acrylic resin in the dough stage). The teeth’ enamel surface was placed above the level of the acrylic resin, where the dentine surface touched the acrylic resin while the enamel surface faced upward.

### Specimen immersions and testing

The selected teeth were assorted into two main groups with 40 samples each. Group DM consisted of the second deciduous molars, while Group PM contained the premolars. Each of the DM and PM groups was then split into two subgroups: Control Subgroup included 20 specimens placed in artificial saliva for 30 days with changing the artificial saliva every day, and Test Subgroup had 20 specimens immersed in a lactoferrin suspension. Lactoferrin sachets used in this study are commercially available under the trade name Pravotin (Green Field for Hygint Pharmaceuticals, Alexandria, Egypt).

The lactoferrin suspension was prepared according to the manufacturer’s instructions. Briefly, 200 mg lactoferrin from two Pravotin sachets were added to 50 ml of water. The mixture was stirred to obtain a 4-mg/ml lactoferrin suspension. Samples in the Test Subgroup were immersed in that suspension for 2 min, then placed in artificial saliva for the rest of the 24 h. This step was repeated for 30 successive days [13].

### The pH measurements

Most pediatric medicaments may contain certain acids in their liquids, which serve as a buffer to keep the solution’s chemical stability and tonicity under control and maintain physiological compatibility. They also enhance the flavor, which makes the drug more kid-friendly [14]. Since this is a pioneering study about the effect of orally supplemented lactoferrin on the enamel exterior of primary and permanent teeth, a pH meter was used to measure the pH of the lactoferrin suspension.

### Surface hardness measurements

Forty-eight specimens (12 specimens from each subgroup) were tested with a Vickers microhardness tester (Tukon 1102, Wilson Microhardness Tester, Buehler, Germany) equipped with a Vickers diamond indenter to investigate the microhardness of both control and test Subgroups. The flattest areas of the enamel surface were indented to guarantee the correctness of the measurements. Three indentations were placed using a diamond indenter by applying a 100 g load for 10 s. Using a microscope connected to the microhardness tester at 50x magnification, the indentation was focused with the magnifying eyepiece, and the two impression diagonals were gauged with a micrometer to the closest 0.1 μm then the mean was calculated. The Vickers Hardness Number (VHN) values were determined after the marking of the diagonals with the VHN software program. The Vickers hardness (HV) was computed using the next formula.

$$HV\, = \,1854.4L/{d^2}$$

Where the load (L) is expressed in gf, the mean diagonal (d) is expressed in m, and the resulting hardness number is demonstrated in gf/m2.

### Surface micromorphological examinations and mineral content measurements

Thirty-two specimens (8 specimens from each subgroup) were removed from the artificial saliva and allowed to dry for two hours. Then, samples were examined using an ESEM (Quanta FEG 250, FEI Company, Netherland) (Desert Research Center, Matarya, Egypt) and EDX to monitor the ultramorphology of the enamel exterior and assess the amounts of calcium and phosphorus (in weight%), as well as the calcium to phosphorus ratio.

ESEM, in conjunction with EDX, delivers detailed high-resolution images of the specimen by rasterizing a focused electron beam across the surface and collecting backscattered electron signals. Environmental microscopy offers a comparatively new option for imaging hydrated materials and does not require prior sample preparation or conductive coating [15].

### Statistical analysis

A normality test (Shapiro-Wilk) was conducted to ensure the data were normally distributed. Data were presented as the Mean ± Standard Deviation (SD).

An independent sample T-test was employed to compare each component of the two groups for all variables. The statistical analysis was accomplished using SPSS software version 26.0 for Windows (IBM Corp, Armonk, NY, USA).

## Results

### pH

The results revealed that the drug under study (Pravotin sachets) had an initial pH ranging from 6.7 to 6.8. The pH of Pravotin suspension gradually decreased until it reached 2.0 on the 4th day and 1.4 on the 6th day (Table 1).

**Table 1:** Change in pH values of lactoferrin suspension

Days	pH
Day.1	6.8
Day.2	5.4
Day.3	4.2
Day.4	2
Day.5	1.7
Day.6	1.4

### Microhardness

The current findings indicated that the lactoferrin subgroup of Group DM experienced a highly statistically significant decrease in microhardness (*p* < 0.01), and the lactoferrin subgroup of Group PM experienced a less statistically significant reduction in microhardness (*p* < 0.05) (Table 2).

**Table 2:** Comparison between microhardness in all groups using T test

		Mean	SD	Min	Max	T	*P* value
Group DM (Deciduous molars)	Control group	316.60	34.29	275.50	382.93	5.11	< 0.001**
	Test group	244.00	35.31	160.10	291.20		
Group PM (Premolars)	Control group	301.92	37.98	228.57	366.03	2.639	0.015*
	Test group	264.62	30.90	186.70	300.13		

* means significant difference between groups at *P* value < 0.05

** means highly significant difference between groups at *P* value < 0.01

### EDX (Mineral content)

Calcium (Ca) and phosphorus (P) weight: There was a decrease in calcium weight% in lactoferrin subgroups in both DM and PM groups; however, the decline was not statistically significant (*p* > 0.05) (Table 3). Also, there was a decrease in phosphorus weight% in lactoferrin subgroups in both DM and PM groups, but with statistical significance (*p* < 0.05) (Table 4). Ca:P ratio showed a non-statistically significant decrease in lactoferrin subgroups in both DM and PM groups compared with Ca:P ratio in the control subgroups (*p* > 0.05) (Table 5).

**Table 3:** Calcium weight percentage of both groups (Deciduous molars) and (Premolars)

Group	Mean	SD	T	*P* values < 0.05
control group of deciduous molars	43.445	5.855	1.250	0.232
Test group of deciduous molars	40.406	3.609		
control group of Premolars	47.730	8.150	0.754	0.464
Test group of Premolars	45.101	5.560		

**Table 4:** Phosphorus weight% of both groups (Deciduous molars) and (Premolars)

Group	Mean	SD	T	*P* values < 0.05
control group of deciduous molars	19.101	0.337	2.208	0.044*
Test group of deciduous molars	18.544	0.629		
control group of Premolars	19.000	0.748	2.495	0.026*
Test group of Premolars	18.258	0.386		

* means significant difference between groups at *P* value < 0.05

**Table 5:** Calcium phosphorus ratio of both groups (Deciduous molars) and (Premolars)

Group	Mean	SD	T	*P* values < 0.05
control group of deciduous molars	2.27	0.334	0.604	0.556
Test group of deciduous molars	2.19	0.0256		
control group of Premolars	2.50	0.342	0.214	0.833
Test group of Premolars	2.46	0.284		

### Environmental Scanning Electron Microscopy

**Fig. 1:**
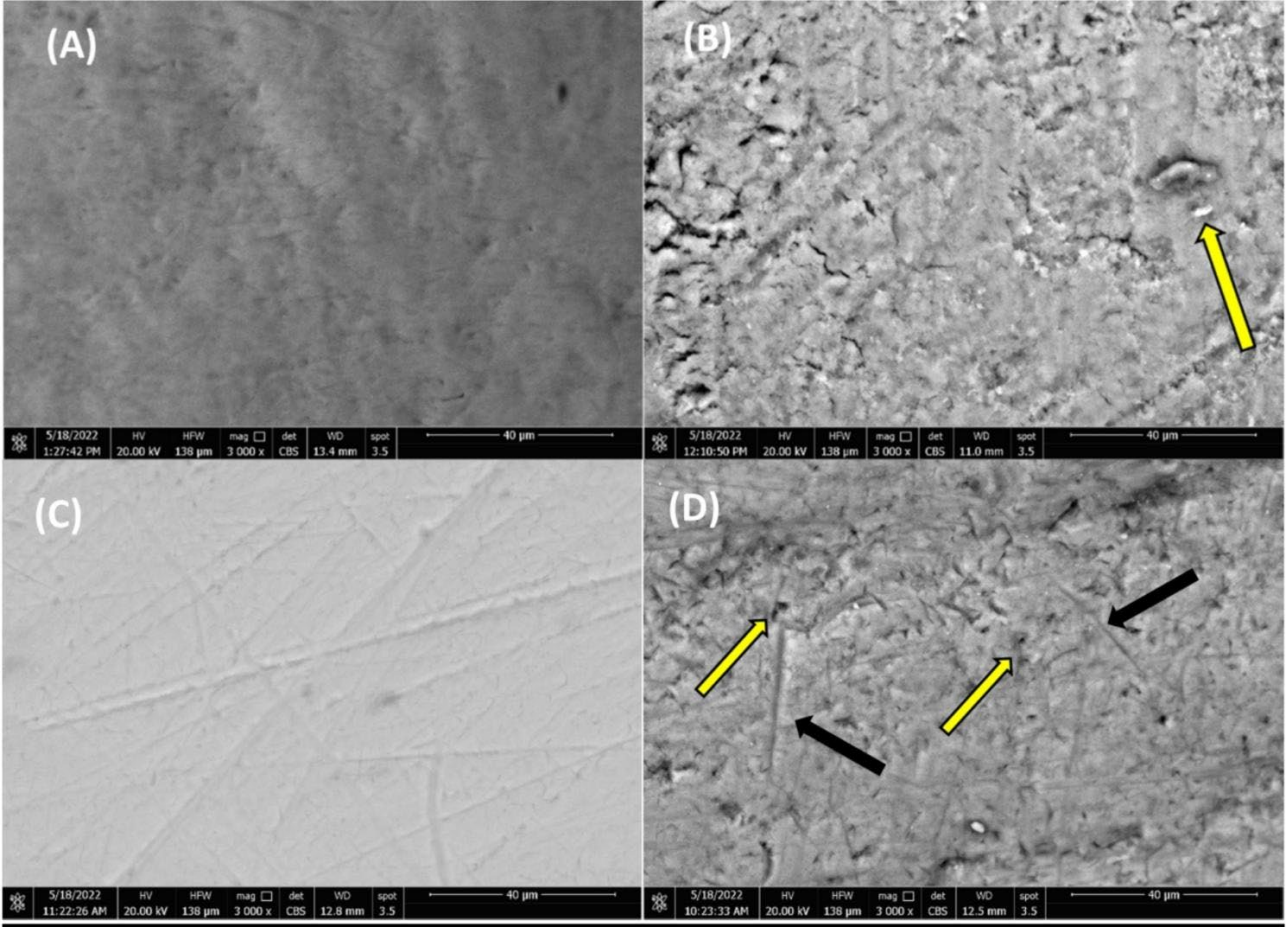
ESEM micrograph of Teeth specimens (mag. X 3000). Before immersion in lactoferrin suspension: (**A**) untreated enamel surface of DM group (Deciduous molars), (**C**) untreated enamel surface of PM group (Premolars). After 30 days of immersion into Lactoferrin suspension: (**B**) treated enamel surface from group DM (Deciduous Molars). The yellow arrow in B photo representing presence of craters with raised rims and some visible dentin, (**D**) treated enamel surface from group PM (premolars) Yellow arrows in D photo representing uneven, pitted, and rough enamel surface. Black arrows representing presence of cracks in enamel surface

The environmental scanning electron micrograph from the control of Group DM exhibited no morphological flaws or signs of degradation, and the enamel surface was intact, somewhat flat, and smooth with characteristic subtle depressions representing rod ends (Fig. 1A). Following the treatment with lactoferrin suspension, the enamel exterior of the primary tooth showed a severely damaged, pitted, rough, and irregular enamel surface with generalized structural loss, as well as several craters with raised rims and some visible dentin. The primary enamel surface was more affected than the permanent one (Fig. 1B).

The scanning electron micrograph of the control from Group PM displayed the normal contour of the enamel surface layer, which was smooth, regular, and homogeneous. Occasional scratch marks and some foci of indefinable debris were noted (Fig. 1C). Treatment of premolars with lactoferrin suspension resulted in demineralized enamel and porous surface, as demonstrated by scanning electron microscopy. The unification of the enamel rods was hardly undermined by an extensive, uneven, pitted, and rough surface, which was visible via magnifying the demineralized enamel prismatic pattern. Also, there were numerous pores of varied sizes and depths on the rough, damaged, and fractured surface (Fig. 1D).

## Discussion

Lactoferrin has multiple effects on the immune and gastrointestinal systems in neonates, babies, and early children, and it is safe to use orally in children [16]. Lactoferrin is an orally supplemented suspension; therefore, it may affect the enamel surface of teeth due to contact with teeth during ingestion.

The specimens used in this study were sound, extracted, primary (second molar), and permanent teeth (first premolar) obtained from 11- to 14-year-old patients. The teeth from patients within such an age range will most likely be removed for shedding and orthodontic causes. This age group was selected to standardize the newly erupted premolars, as there may be a difference in the composition of the enamel surface between the newly erupted teeth and older teeth [17]. Teeth specimens proved to be free of cracks, cavities, restorations, or morphological anomalies when examined with a dental operating microscope, as it enhances the identification of cracks [18], eliminating the possibility of any effect of the naturally occurring changes on the results of the microhardness test [19].

The teeth were meticulously cleaned and autoclaved at 121 °C for 20 min, according to the guidelines of the United States Center for Disease Control (CDC). Autoclaving achieves 100% efficacy in sterilizing extracted teeth [20], and it doesn’t affect the characteristics of tooth enamel [21]. Then, the teeth were kept at room temperature in normal saline for no longer than one month to prevent dehydration, as extended storage times may impair the mineral composition of enamel [22]. The storage medium was changed every day to prevent bacterial growth. The teeth were then cut in a level of the cementoenamel junction using a sharp disc with coolant to avoid tooth cracking and then were divided mesio-distally for standardization.

A 3D-printed resin block made by the CAD-CAM technology was utilized to construct a rubber mold to fabricate cold-cure resin blocks. All the resin blocks cast in this rubber mold had standardized flat bases. Samples were tested with a Vickers Micro Hardness Tester and then inserted into these cold-cure resin blocks, which have flat bases to apply perpendicular force on the tested surface.

Artificial saliva was employed as a control subgroup and placement medium for specimens in the test subgroup (lactoferrin subgroup) to mimic the oral cavity’s environment and supply natural nutrients like calcium and phosphorus [23, 24]. The artificial saliva in the current study had a pH of 7.4, which falls in the normal range of natural saliva (6.2–7.6) [25], to eliminate the effects of pH differences between natural and experimental conditions.

Because the typical time to consume the suspension at room temperature is about two minutes, the current study was undertaken by submerging the specimens in a lactoferrin suspension for that duration. In addition to lactoferrin, the surface hardness of the teeth can also be influenced by saliva. The current research was carried out at room temperature to lessen the impact of temperature on degradation [26]. Given that taking bovine lactoferrin orally for thirty days dramatically raises serum ferritin, hemoglobin, total iron, and red blood cell counts [5], the treatments in this study continued for 30 days.

According to our results, the pH of lactoferrin suspension was initially between 6.7 and 6.8, and it gradually dropped over time. This is comparable to the majority of pediatric liquid drugs, which have a significant potential for erosive damage since they contain an acid in their formula. The pH range of routinely advised pediatric liquid medications (PLM) was 4.22 to 6.10. Sucrose is also present in the PLM and in the samples in concentrations ranging from 5.38 to 11.41 gm%; thus, they have the potential to cause caries [7].

Each sachet of Pravotin contains 100 mg lactoferrin and other components, including lactose anhydrous, sucralose, maize starch, magnesium stearate, and orange flavor. The suspensions of lactoferrin have a higher viscosity than water, exhibit low solubility in water, adhere to the enamel surface of the samples, and are difficult to clean. Our specimens were immersed in Pravotin suspension for two minutes, then placed in artificial saliva without washing for the rest of the 24 h for 30 successive days. In conclusion, the longer the exposure of tooth enamel to sugar, starch, or acid, the more demineralization [27].

According to the findings of this investigation, specimens of Group DM exposed to the lactoferrin suspension displayed a highly significant reduction in microhardness in contrast to the control subgroup, and the samples of Group PM subjected to the lactoferrin suspension indicated a significant reduction in microhardness relative to the control subgroup. This decrease in microhardness may be due to the decline in pH of the lactoferrin suspension since pediatric liquid syrups with acidic pH reduce enamel microhardness [28, 29]. Iron and multivitamin drops can cause erosion and decrease the enamel microhardness of primary teeth due to low pH, as the pH of the used drops was in the range of 2.1–3.3, which falls below the enamel’s critical pH range [30]. This reduction in microhardness may also be due to the sugar content of the drug, as sugar-containing syrups cause a reduction in the microhardness of enamel regardless of their pH values [29, 31].

Regarding the EDX results, specimens treated with lactoferrin in both DM and PM groups revealed that the decline in Ca weight% was not statistically significant compared with a statistically significant reduction in the P weight%. Ca/P ratio also decreased with no statistical significance, explaining the decline in microhardness, which may be due to the breakdown of hydroxyapatite and loss of mineral content (calcium and phosphorus) [32]. Demineralization of enamel arises from chemical reactions that cause the major enamel component, hydroxyapatite crystals, to break down. This procedure happens when the enamel surface’s surrounding fluid has a pH lower than 5.5 [33]. Thus, the effect of lactoferrin suspension on tooth mineral content (calcium and phosphorus) may be due to its acidic nature and the presence of sucrose in its composition [34].

Environmental microscopy offers a comparatively new technique for visualizing hydrated materials and does not require sample preparation or conductive coating [15]. As shown by the current ESEM results, the specimens of Groups DM and PM treated with lactoferrin showed demineralized and porous enamel surfaces. The unification of the enamel rods was seriously compromised by significant pitting, as revealed by a close inspection of the prismatic pattern of demineralized enamel under extreme magnification (3000x). The enamel exterior of primary teeth (Group DM) was more affected than that of permanent teeth (Group PM), considering the difference in their enamel thicknesses, mineralization levels, and structural arrangement [35]. The inorganic compounds comprise roughly 92% of permanent enamel and 86–88% of primary enamel, explaining the profound effect on primary enamel than on permanent enamel [36], which may be due to the drop in pH below the critical point [30, 34]. The scanning electron microscope image demonstrated the ability of pediatric liquid medication syrups with high sugar content and viscosity to destroy both primary and permanent teeth, even when the pH is above the critical point. Additionally, highly viscous acidic sugar-free formulations with high titratable acidity and buffering capacity may retain an erosive effect [31, 37].

This study aimed to mimic the influences of taking lactoferrin suspension orally; however, certain in vivo conditions need to be considered, including the pH and temperature variations in the oral cavity, which may affect the findings of the current study. Additional studies should evaluate the impact of lactoferrin suspension on enamel characteristics for a longer duration in the in vitro state, considering that daily meals and beverages consumed in vivo may influence the outcomes. Also, more experiments are required to assess the effect of lactoferrin suspension in vivo.

## Conclusions

Lactoferrin (Pravotin) suspension led to a significant decrease in microhardness of enamel (*p* < 0.05), a significant reduction in phosphorus weight percentages (*p* < 0.05) and areas of erosion in enamel surface in both dentitions but primary teeth were more affected than the permanent teeth.

## Data Availability

Upon a reasonable request, the corresponding author can provide the datasets utilized and/or generated in the current research.

## Abbreviations

EDX: Energy dispersive X-ray spectroscopy
ESEM: Environmental scanning electron microscopy
pH: Potential of hydrogen
SD: Standard deviation
PLM: Pediatric liquid medication
3D printed: 3 Dimensional printed block
